# Clinical outcomes of ocular surface in patients treated with vitamin D oral replacement

**DOI:** 10.5935/0004-2749.20200043

**Published:** 2020

**Authors:** Emine Esra Karaca, Özlem Evren Kemer, Dilay Özek, Dilek Berker, Narin Nasiroglu Imga

**Affiliations:** 1 Department of Ophthalmology, Ankara Numune Training and Research Hospital, Health Sciences University, Ankara, Turkey; 2 Department of Endocrinology and Metabolism, Ankara Numune Training and Research Hospital, Health Sciences University, Ankara, Turkey

**Keywords:** Dry eye syndrome, Vitamin D deficiency, Dietary supplements, Síndromes do olho seco, Deficiência de vitamina D, Suplementos nutricionais

## Abstract

**Purpose:**

To analyze the clinical outcomes of the ocular surface in patients with vitamin D deficiency after oral replacement.

**Methods:**

A total of 40 patients with vitamin D deficiency were enrolled in the study. The patients received 50,000 units of oral vitamin D weekly over a period of 8 weeks. After 8 weeks, 1,500-2,000 units/d were administered for 24 weeks. Eyelid margin score, meibomian gland expressibility score, Oxford grading, Schirmer I test, tear breakup time, tear osmolarity, and the Ocular Surface Disease Index score were evaluated at baseline, and at 8, 12, and 24 weeks.

**Results:**

The meibomian gland expressibility score, Schirmer I, tear breakup time, tear osmolarity, and Ocular Surface Disease Index score showed improvement 8 weeks after vitamin D supplementation (p<0.05). Compared with the pretreatment values, the eyelid margin score and Oxford grading were decreased at week 12 (p<0.05).

**Conclusion:**

Vitamin D replacement appears to improve ocular surface in individuals with vitamin D deficiency.

## Abstract (pt)

**Objetivo:**

Analisar os resultados clínicos da superfície ocular em pacientes com deficiência de vitamina D após reposição oral.

**Métodos:**

Foram incluídos no estudo 40 pacientes com deficiência de vitamina D. Os pacientes receberam 50.000 unidades de vitamina D semanalmente por um período de oito semanas. Após esse período, 1.500-2.000 unidades/dia foram administradas por 24 semanas. Escores da margem palpebral, escores de expressibilidade da glândula meibomiana, classificação de Oxford, teste de Schirmer I, tempo de ruptura lacrimal, osmolaridade da lágrima e escore do Índice de Doenças da Superfície Ocular foram avaliados no início e após 8, 12 e 24 semanas.

**Resultados:**

O escore de expressibilidade da glândula meibomiana, Schirmer I, tempo de ruptura lacrimal, osmolaridade da lágrima e o Índice de Doenças da Superfície Ocular apresentaram melhoras após 8 semanas de suplementação de vitamina D (p<0,05). Comparado com os valores do pré-tratamento, o escore da margem palpebral e a classificação de Oxford diminuíram na 12ª semana (p<0,05).

**Conclusão:**

A reposição de vitamina D parece melhorar a superfície ocular em indivíduos com deficiência de vitamina D.

## INTRODUCTION

Vitamin D, produced in the skin following exposure to sunlight, is a fat-soluble vitamin. It plays vital roles in cartilage and bone metabolism^(1)^, as well as immunomodulation^(2)^. Vitamin D and the vitamin D receptor regulate genes that contribute to inflammation, immunity, and cellular proliferation^(3)^. Vitamin D deficiency, a common health problem worldwide, may cause ocular diseases, such as myopia, age-related macular degeneration, diabetic retinopathy, uveitis, and dry eye syndrome (DES)^(4,5)^.

Ocular inflammation and increased osmolarity are the leading problems in patients with DES^(6)^. DES results in discomfort, visual disturbance, and tear film stability that damages the ocular surface^(6)^. It is assumed to be a localized autoimmune disease. Recently, it was found to be related to vitamin D deficiency because of its anti-inflammatory action^(7-9)^. DES exerts a negative effect on quality of life, and patients with DES mostly complain of chronic ocular fatigue and pain^(10)^.

Vitamin D supplementation has been widely used for the treatment of several diseases. It has been reported to strengthen immunity, relieve inflammation, and regulate the cell cycle^(11-13)^. Recently, vitamin D was suggested to play a role in modulating corneal wound healing and enhancing the function of the corneal epithelial barrier^(14,15)^.

Patients with vitamin D deficiency are typically followed up in endocrinology clinics, and most of them are unaware of the eye-related complaints. For this reason, we aimed to investigate the ocular surface health in patients with vitamin D deficiency, evaluate their tendency toward DES, and demonstrate the effect of treatment with vitamin D on the ocular surface.

## METHODS

The study was performed in adherence to the tenets of the Declaration of Helsinki and approved by the local ethics committee. Forty patients (aged >18 years), newly diagnosed with vitamin D deficiency (serum 25-hydroxyvitamin D levels <20 ng/mL) in an endocrinology and metabolism outpatient clinic, were enrolled in this study. The mean age of the patients was 48.4 ± 11.08 years. There were 34 women and 6 men. Patients with meibomian gland disease, with or without DES were included. However, patients with any serious systemic disease (e.g., primary Sjögren’s syndrome) or other systemic rheumatic disease history, vitamin B12 deficiency, pregnancy, breastfeeding, history of smoking, current drug use, active ocular infection or allergy, previous eye surgery, and use of contact lenses were excluded.

Prior to the initiation of vitamin D supplementation, the patients underwent a complete ophthalmological examination. The patients received 50,000 units of oral vitamin D weekly, over a period of 8 weeks. After 8 weeks, 1,500-2,000 units/d were administered for 24 weeks^(16)^. The eyelid margin score (LMS), meibomian gland expressibility score (MGS), Oxford grading, Schirmer I test, tear breakup time (TBUT), tear osmolarity, and Ocular Surface Disease Index (OSDI) score were evaluated at baseline, and at 8, 12, and 24 weeks. The patients were maintained at 21°C and in 40% humid environment for 1 h while completing the OSDI questionnaire. All measurements were performed by one examiner with same order after waiting patients in the same room (E.E.K.). The right eyes of the patients were examined.

LMS was evaluated as follows: eyelid margin irregularity (presence/absence), vascularity of the eyelid mar gin (presence/absence), occlusion of glands at the lid margin (presence/absence), and displacement of the mucocutaneous junction (presence/absence), scored on a 0-3 scale. MGS was interpreted in accordance with the quality of meibomian gland secretion, scored on a 0-3 scale (grade 0: clear meibum, easily expressed; grade 1: cloudy meibum, easily expressed; grade 2: cloudy meibum, expressed with moderate pressure; grade 3: meibum not expressible, even with hard pressure)^(17)^.

The Schirmer I test was performed by placing a 5 × 35 mm strip of standard filter paper in the lower eyelid one-third of the distance from the lateral canthus and recording the wetted distance (in mm) after 5 min. TBUT was evaluated by examining the fluorescein-stained tear film with a biomicroscope using cobalt blue light and measuring the time between a blink and the first appearance of a dry spot. After staining with fluorescein, corneal punctate erosion staining was recorded using the standardized Oxford grading system^(18)^. Measurement of tear osmolarity was conducted using a TearLab osmometer (TearLab Corp, San Diego, CA, USA). Tears were collected from the inferior lateral tear meniscus. Three consecutive measurements were obtained, and their mean was used for further evaluation.

The OSDI questionnaire consists of three main sections concerning ocular symptoms, visual function, and environmental factors^(19)^.

### Statistical analysis

All data are presented as mean ± standard deviation. Paired-sample t-tests were used to compare the eyelid margin, meibomian gland expressibility score, Oxford grading, Schirmer I test, TBUT, tear osmolarity, and OSDI score at baseline, and 8, 12, and 24 weeks after vitamin D supplementation. The SPSS version 22 for Windows (IBM Corp., Armonk, NY, USA) software was used for all analyses. A p<0.05 denoted statistical significance. With the 40 patients enrolled in this study, we had 80% power to detect an effect size (W) of 0.714 using a two-degree of freedom chi-squared test with α=0.05.

## RESULTS

**Figure 1:**
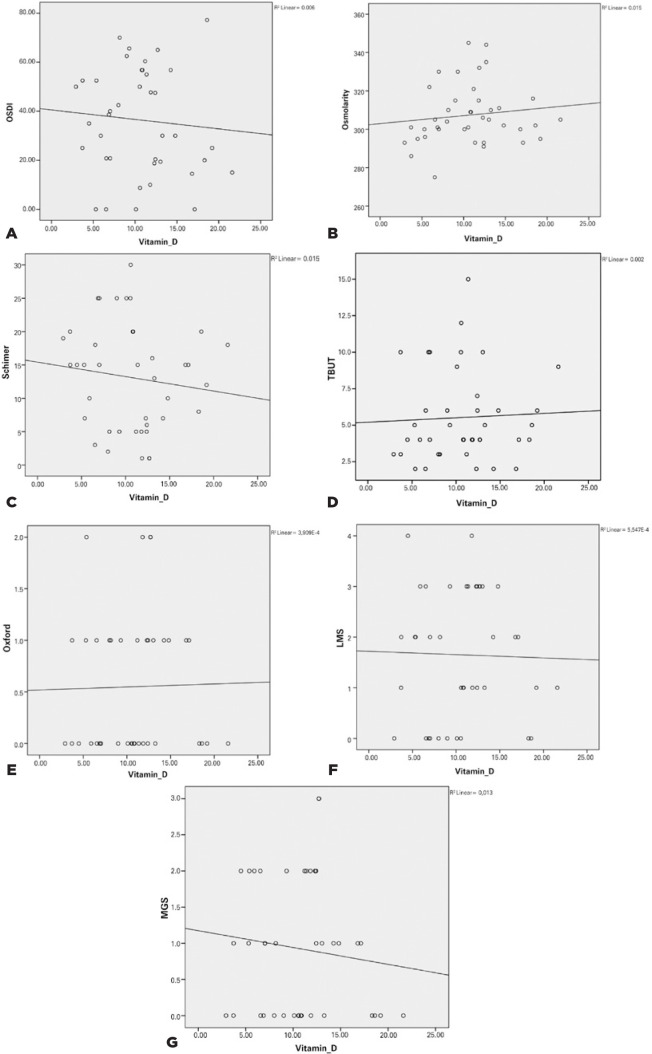
Correlation graphs showing the levels of vitamin D and tear function tests.

The mean levels of 25(OH)D in the serum at baseline were 10.71 ± 4.59 ng/mL. The correlation between the levels of vitamin D and LMS, MGS, Oxford, Schirmer I, TBUT, tear osmolarity, and OSDI is shown in figure 1. The effects of treatment with vitamin D on ocular surface parameters were evaluated (Table 1). MGS also improved after treatment compared with its level at baseline (p<0.05). The Oxford grading and eyelid margin scores decreased significantly after 12 weeks of treatment (p<0.05). The Schirmer I test score increased from 13.10 ± 8.01 mm at baseline to 17.33 ± 7.29 mm after 24 weeks (p=0.002, p<0.001, and p<0.001, respectively). TBUT improved from 5.53 ± 3.12 s to 9.13 ± 3.01 s (all p<0.001). Tear osmolarity was 307.4 ± 15.4 mOsm/L at baseline and 302.7 ± 10.6 after 24 weeks (all p<0.001). The OSDI score improved throughout the entire study period (all p<0.001).

**Table 1:** The effects of treatment with vitamin D on ocular surface parameters

	Baseline Mean ± SD	After vitamin D supplementation					
		8 weeks		12 weeks		24 weeks	
		Mean ± SD	p-value	Mean ± SD	p-value	Mean ± SD	p-value
Eyelid margin score	1.65 ± 1.27	1.60 ± 1.24	0.160	1.33 ± 1.02	0.000^*^	1.28 ±1.01	0.000^*^
Meibomian gland expressibility score	0.93 ± 0.94	0.83 ± 0.81	0.044^*^	0.73 ± 0.78	0.003^*^	0.70 ± 0.79	0.005^*^
Oxford grading	0.55 ± 0.68	0.43 ± 0.59	0.058	0.33 ± 0.57	0.002^*^	0.33 ± 0.57	0.002^*^
Schirmer 1 tear secretion test (mm)	13.10 ± 8.01	15.03 ± 7.80	0.002^*^	16.55 ± 7.26	0.000^*^	17.33 ± 7.29	0.000^*^
TBUT (s)	5.53 ± 3.12	6.90 ± 2.72	0.000^*^	8.30 ± 2.66	0.000^*^	9.13 ± 3.01	0.000^*^
Tear osmolarity	307.4 ± 15.4	304.1 ±11.8	0.000^*^	303.5 ± 12.1	0.000^*^	302.7 ± 10.6	0.000^*^
OSD1 score	36.4 ± 22.1	27.40 ± 18.2	0.000^*^	21.97 ± 13.9	0.000^*^	19.12 ± 11.9	0.000^*^

* p<0.05 by paired t-test compared with baseline.

TBUT= tear breakup time; OSDI= Ocular Surface Disease Index; SD= standard deviation.

(OSDI= Ocular Surface Disease Index; TBUT= tear breakup time; LMS= eyelid margin score; MGS= meibomian gland expressibility score).

## DISCUSSION

In this study, we assessed the ocular surface health of patients with vitamin D deficiency and investigated the effects of treatment with vitamin D on tear function and the ocular surface. The tear function and ocular surface health of these patients showed improvement during the treatment.

Vitamin D regulates the levels of calcium and phosphate in the serum, thereby exerting an important effect on bone health. Furthermore, vitamin D deficiency is closely related to certain autoimmune diseases, such as rheumatoid arthritis, systemic lupus erythematosus, multiple sclerosis, type I diabetes mellitus, and inflammatory bowel diseases^(20,21)^. Hence, vitamin D supplementation is recommended in the treatment of certain rheumatic diseases^(22)^. Numerous studies showed an association between vitamin D and inflammatory markers. Vitamin D showed a negative relation with the levels of C-reactive protein and interleukin 6 (IL-6)^(23)^. In another study, vitamin D was suggested to induce the production of IL-10, which inhibits the production of certain proinflammatory cytokines (e.g., IL-1, IL-6, and tumor necrosis factor-α)^(24)^.

DES is assumed to be a localized autoimmune disease. Thus, current studies presume that vitamin D plays an important role in DES and ocular surface health, owing to its anti-inflammatory properties^(7,25)^. In addition, vitamin D induces cathelicidin produced by corneal and conjunctival epithelial cells and assists corneal and conjunctival wound healing^(26,27)^. Yin et al. reported the presence of the vitamin D receptor and vitamin D metabolites in corneal epithelial samples^(15)^. They claimed that 25(OH)D(3) and its active metabolite 1,25(OH)(2)D(3), enhanced the function of the corneal epithelial barrier. Thus, vitamin D may play a role in the development of DES and wound healing. Yildirim et al. showed that patients with vitamin D deficiency developed DES and showed impaired tear function^(28)^. They demonstrated lower scores in the Schirmer I test and TBUT, and higher in the OSDI score in vitamin D deficient patients versus controls. Demirci et al. reported that the TBUT score and Schirmer I test results were significantly lower in patients with vitamin D deficiency versus the control group^(5)^. Tear osmolarity values, and the OSDI and Oxford grading scores were significantly higher than those reported in the control group. Osmolarity is one of the most objective parameters of DES and contributes to the pathogenesis of ocular surface damage^(7)^. Osmolarity aggravates tear film instability and negatively affects the ocular surface. In our study, tear osmolarity at baseline was significantly higher than that observed post treatment. However, there was no significant correlation between the levels of vitamin D and osmolarity.

Recent studies established the relationship between vitamin D deficiency and Sjögren’s syndrome^(7,29)^. Bang et al. reported lower levels of vitamin D in patients with Sjögren’s syndrome^(30)^. In addition, they revealed a relationship between Sjögren’s syndrome activity and the levels of vitamin D. On the contrary, we did not find an association between the levels of vitamin D and TBUT, Schirmer I, Oxford grading scale, MGS, LMS, and tear osmolarity values. However, following vitamin D replacement, all these parameters showed significant improvement. Recently, the mechanisms through which diet, hormones, and habits influence ocular surface health and tear production are a major concern among ophthalmologists. The use of omega-3 fatty acids has been recommended by the American Academy of Ophthalmology Preferred Practice Pattern guidelines for relieving symptoms and signs in DES patients, despite the lack of evidence. Hence, the Dry Eye Assessment and Management (DREAM) trial investigated the mean change in OSDI, conjunctival staining, corneal staining, TBUT, and Schirmer’s test scores of patients with DES receiving n-3 fatty acid or olive oil placebo at 6 and 12 months^(31)^. The results did not show improvements in these parameters in patients receiving n-3 fatty acids or placebo. In addition, patients in the DREAM study were selected according to the following criteria: presence of conjunctival and corneal staining, TBUT <8 s, Schirmer I test 1-7 mm, and OSDI score 25-80. Although osmolarity is an important finding in DES, the investigators did not report changes in this parameter during active omega-3 supplementation.

The DEWS II report defined DES as “a multifactorial disease of the ocular surface characterized by a loss of homeostasis of the tear film, and accompanied by ocular symptoms, in which tear film instability and hyperosmolarity, ocular surface inflammation and damage, and neurosensory abnormalities play etiological roles”^(32)^. Moreover, this new report emphasized the role of inflammation in DES. It is established that vitamin D is a fat-soluble vitamin and possesses anti-inflammatory properties. The ocular surface health of the patients in the present study (TBUT, Oxford, LMS, MGS, osmolarity) improved after vitamin D replacement, on account of its anti-inflammatory properties and effects on the function of the epithelial barrier. It is proposed that vitamin D does not affect the secretion capacity of the lacrimal glands. Therefore, the results of the Schirmer I test were approximately within the normal range of values and showed limited improvement after oral replacement.

This study had some limitations, such as the small sample size and lack of a control group. Patients who were not deficient in vitamin D were not included as a control group in this study. Additionally, we did not investigate the presence of inflammatory markers in the patients. Tear osmolarity, OSDI, and other tear function parameters appear to have improved following vitamin D supplementation.

In conclusion, vitamin D replacement appears to improve ocular surface health in patients with vitamin D deficiency. However, further clinical trials with a large sample size and control group are warranted to define the role of vitamin D.
